# Life-threatening urosepsis because of ureteral entrapment within a large inguinal hernia

**DOI:** 10.1093/jscr/rjad589

**Published:** 2023-10-25

**Authors:** Alexandros Chamzin, George Galyfos, Konstantinos Saliaris, Maximos Frountzas, Panagiotis Theodorou, Dimitrios Theodorou

**Affiliations:** First Propaedeutic Department of Surgery, National and Kapodistrian University of Athens, Hippokration General Hospital, 114 Vasilissis Sofias Avenue, Athens 11527, Greece; First Propaedeutic Department of Surgery, National and Kapodistrian University of Athens, Hippokration General Hospital, 114 Vasilissis Sofias Avenue, Athens 11527, Greece; First Propaedeutic Department of Surgery, National and Kapodistrian University of Athens, Hippokration General Hospital, 114 Vasilissis Sofias Avenue, Athens 11527, Greece; First Propaedeutic Department of Surgery, National and Kapodistrian University of Athens, Hippokration General Hospital, 114 Vasilissis Sofias Avenue, Athens 11527, Greece; First Propaedeutic Department of Surgery, National and Kapodistrian University of Athens, Hippokration General Hospital, 114 Vasilissis Sofias Avenue, Athens 11527, Greece; First Propaedeutic Department of Surgery, National and Kapodistrian University of Athens, Hippokration General Hospital, 114 Vasilissis Sofias Avenue, Athens 11527, Greece

**Keywords:** inguinal hernia, ureteral entrapment, sepsis

## Abstract

Ureteral hernias are an uncommon entity that are usually incidentally discovered during inguinal hernia repair. However, when symptomatic, they could cause severe symptoms from the urinary system and even affect renal function. We aim to report a rare case of a 91-year-old male patient with urosepsis because of ureteral entrapment within an inguinal hernia, and further discuss proper management of such cases.

## Introduction

Inguinal hernia repair is one of the most frequent surgical procedures, with ~20 million repairs performed worldwide annually [1]. Common complications of a hernia requiring surgery include incarceration, bowel obstruction, and strangulation [1]. However, an inguinal hernia containing a ureter is an uncommon clinical entity and usually is asymptomatic [2]. Ureteral hernias were first reported in the nineteenth century, but since then only about 150 cases have been reported in the literature [3]. An inguinal hernia may contain the bladder more often, presenting with dysuria, frequency, urgency, and two-stage urination. When the ureter is entrapped, manifestations can include flank pain, recurrent urinary tract infections, or chronic renal damage because of ureteral obstruction. In cases of prolonged entrapment, hydronephrosis and even sepsis may occur [2].

Therefore, we aim to describe a rare case of urosepsis because of ureteral entrapment in a large inguinal hernia and discuss on proper management.

## Case presentation

A 91-year-old patient with an indwelling urinary catheter presented to the Emergency Department complaining about flank pain, a feeling of pressure in the suprapubic area and fever (temperature = 39°C). His past medical history included benign prostate hyperplasia, type II diabetes mellitus, and arterial hypertension. There was no history of prior abdominal surgery. Physical examination revealed a soft, non-tender abdomen, with a non-tender, non-reducible right inguinal hernia. Laboratory workup was significant for marked leukocytosis (white cell count 22 × 103/ml), increased inflammatory markers, and elevated blood urea and creatinine. Ultrasonographic examination revealed a pelvicalyceal dilatation of the right kidney. The patient was admitted to the hospital for antibiotic treatment and further investigation.

**Figure 1:**
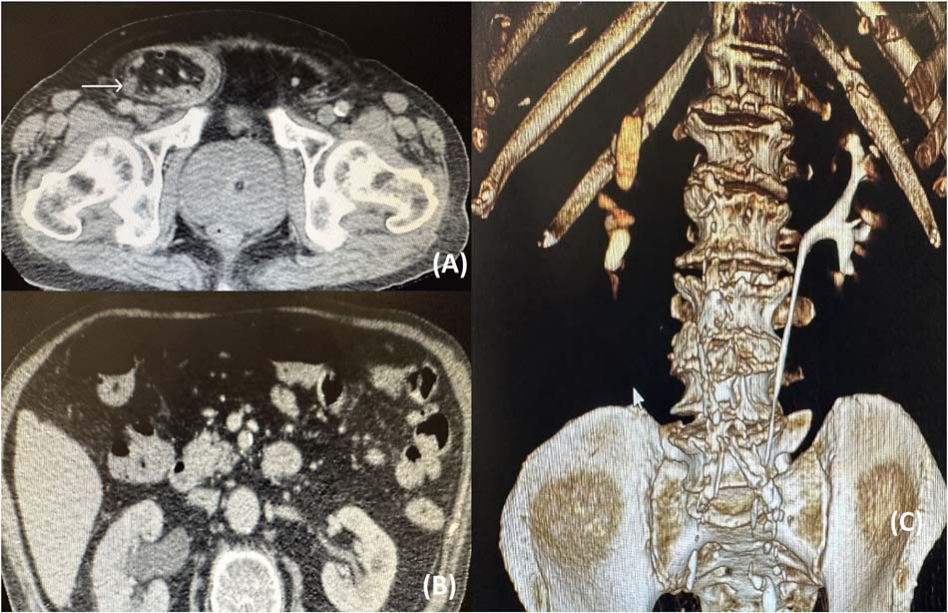
(A) CT image showing the right ureter (arrow) entrapped within the hernia; (B) CT image showing the pelvicalyceal dilatation of the right kidney because of the distal ureteral entrapment; (C) CT reconstruction showing no illustration of the right ureteral system.

A computed tomography (CT) was ordered that revealed a significant pelvicalyceal dilatation and hydronephrosis of the right kidney consistent with obstructive uropathy and a medium-sized right inguinal hernia containing fat, bowel loops, and the right ureter (Fig. 1).

**Figure 2:**
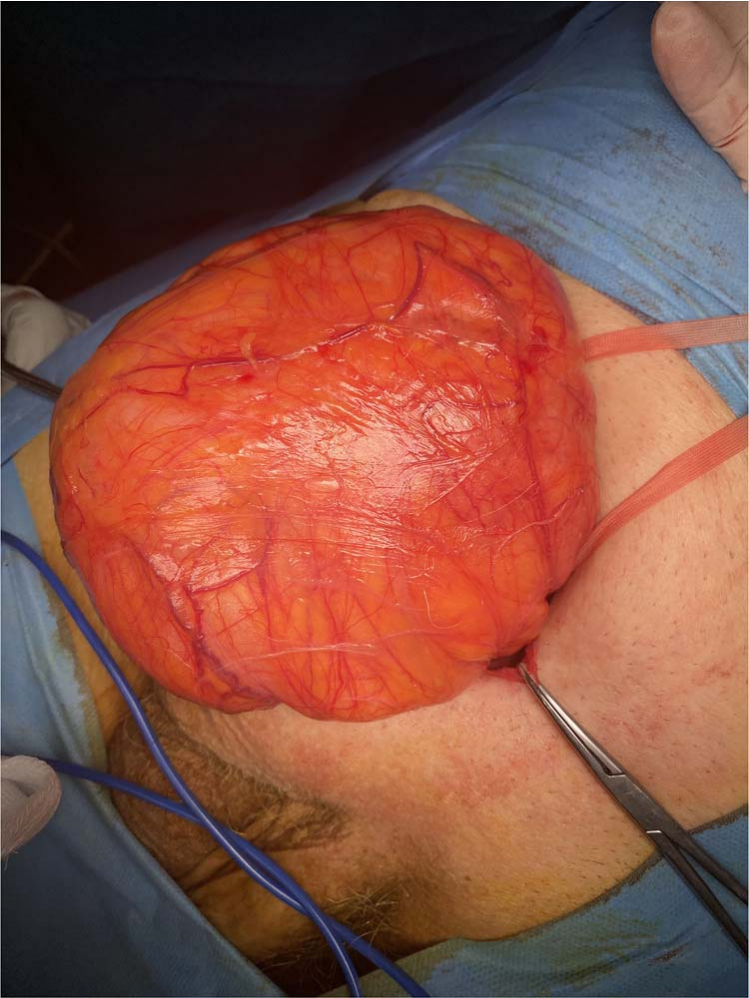
Intraoperative image showing the large hernia containing a large amount of retroperitoneal fat.

**Figure 3:**
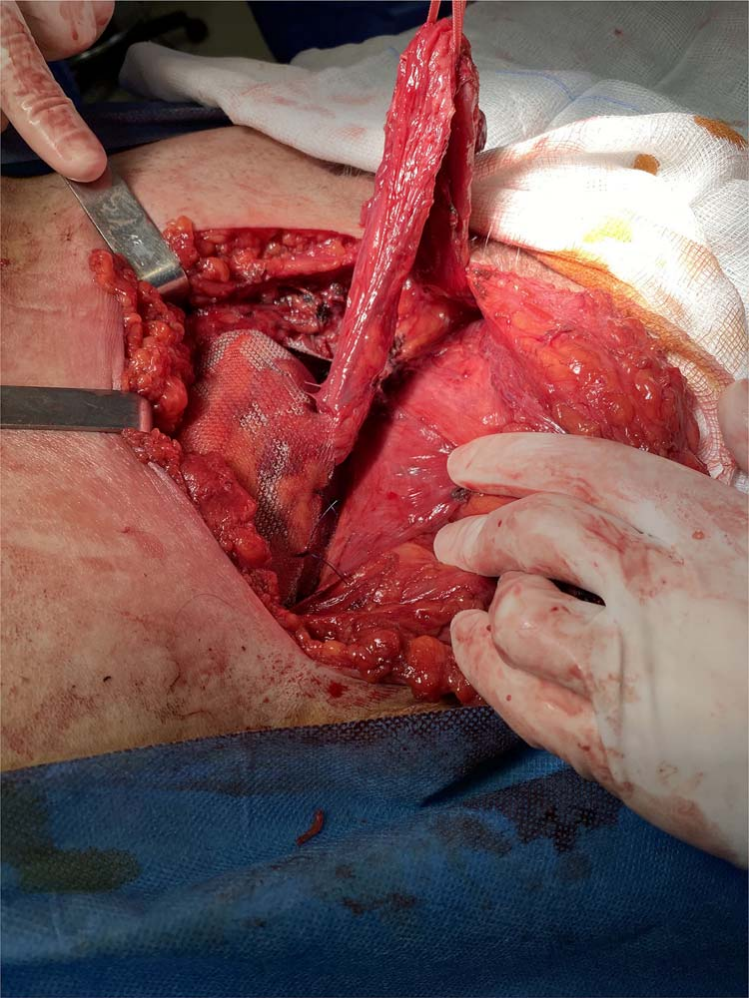
Intraoperative image showing the final reconstruction after mesh placement.

Following multidisciplinary consultation, a stent placement was considered too dangerous for ureteral injury because of acute angulation. Therefore, a primary repair of the inguinal hernia was decided. Because of the patient’s comorbidities, an open approach was favored. Intraoperative dissection revealed an indirect hernia with protrusion of a peritoneal sac containing small bowel loops and large amount of retroperitoneal fat (Fig. 2). The hernia sac containing the bowel loops and ureter was reduced without opening the sac to avoid iatrogenic injury of the ureter. The hernia was repaired with mesh placement according to the Lichtenstein technique (Fig. 3). The postoperative course was uneventful, with resolution of fever and gradual decrease of inflammatory markers. The patient was discharged safely on the fourth postoperative day, with complete resolution of hydronephrosis and normal renal function. Within a 6-month follow-up, no other complication was reported.

## Discussion

We reported a rare case of inguinal hernia entrapping the ureter and causing hydronephrosis and urosepsis.

Ureteral inguinal hernias more commonly occur in men during the fifth and sixth decades of their life although our patient was over 90 years old [4]. Ureteral herniation is a rare occurrence, and only case reports have been published including mainly patients with an orthotopically transplanted kidney, patients with pelvic tumors, patients with ectopic kidneys, patients with renal pelvic fractures causing ureterosciatic herniation, and others [5]. In some cases, however, no specific causative factor is recognized such as in our case. These hernias occur predominantly on the right side because, on the left side, the fascia of Toldt sits at the root of the sigmoid colon mesentery, which fixes the ureter in the retroperitoneum [6]. This concurs with our case that had a right-sided hernia.

Additionally, ureteral hernias are predominantly indirect (almost 80% of cases), and incarceration is relatively infrequent because of the hernia’s large size [3]. Direct inguinal hernias are more common in older men, develop medially to the inferior epigastric vessels through Hesselbach’s triangle, and are thought to be related to the decreasing abdominal wall tissue strength that occurs with aging [7]. In contrast, indirect hernias are more possible to cause strangulation of the retroperitoneal structures such as the ureter, concurring with our case [8]. There has been, however, a report of a direct hernia causing ureteral obstruction and urosepsis as well [2].

Ureteral hernias are of two anatomical types. A paraperitoneal type, where the ureter slides beside a peritoneal sac and constitutes part of the hernia wall, occurs in 80% of cases. Less commonly an extraperitoneal type occurs (20%), where the ureter is accompanied only by retroperitoneal fat and no peritoneal sac is present [9]. Paraperitoneal hernias are usually acquired although extraperitoneal hernias are associated with an embryologic defect [4]. Patients with ureteral herniation are often asymptomatic, and strangulation is rare given the wide fascial defect in these types of hernias. Symptomatic patients often present with a painless groin swelling, which is more evident during upright and physical activities and characteristically resolves when the patient lies down. Often, the patient experiences groin pain, especially during strenuous physical activity or weight lifting. When the ureter is involved, symptoms from the urinary system such as flank pain and urinary tract infection may occur, like in our case. The morbidity of strangulated inguinal hernias is high, with a perioperative complication rate of 12% and a mortality rate of 6% [10]. Bladder involvement is very rare as it is associated with direct hernias and usually presents with symptoms of bladder outlet obstruction such as urinary retention, frequency, and haematuria [6]. In our case, there was no bladder herniation as it was an indirect hernia.

Certain conditions, such as kidney transplants and obesity, increase the risk of developing these hernias. The tortuous nature of the ureter in ectopic kidneys makes them more susceptible to herniation and potentially obstruction. There have been cases with bilateral ureteral obstruction because of inguinal hernia as well [8]. However, regardless of the etiology, surgical repair is recommended in all cases to decrease the risk of obstructive uropathy. The surgical approach in treating these hernias usually consists of ureteral protection with cystoscopy and stent placement, followed by open repair and reduction. However, when there is a significant angulation of the ureter, preoperative stent placement is not always feasible, like in our case. Other means such as the use indocyanine green stain may help identify the ureter intraoperatively. The laparoscopic approach has been previously described in the management of urinary bladder hernias, but there are only case reports describing the laparoscopic repair of ureteral hernias [4]. Many cases of ureteral herniation are identified incidentally during the open repair of an inguinal hernia. In such a repair, if the hernia contains fat that does not meet the characteristics of a cord lipoma and lacks the presence of the classical peritoneal sac, the possibility of herniating retroperitoneal structures, particularly the ureter, should be raised. In this situation, careful reduction of the fat should be performed. If the fat is completely irreducible, careful dissection at the level of the deep ring should be sought to avoid damaging a ureter. Yahya *et al*. [6] have proposed an algorithm for identifying such hernias and reducing the risk for injuries.

## Conclusion

Ureteral inguinal hernias comprise a significant challenge for the average surgeon. Although rare, clinical suspicion should be high in patients with both signs of obstructive uropathy and an inguinal hernia. Minimally invasive approaches can be challenging and should be attempted only by experienced surgeons. Preoperative ureteral stenting is recommended when feasible. An open repair is a safe option but careful sac dissection or intraoperative use of indocyanine green stain is necessary to avoid ureteral injury.

## Data Availability

All data can be requested through the corresponding author (alex.chamzin@gmail.com).

## References

[1] Simons MP , Aufenacker T, Bay-Nielsen M. et al. European hernia society guidelines on the treatment of inguinal hernia in adult patients. Hernia 2009;13:343–403. 19636493 10.1007/s10029-009-0529-7 PMC2719730

[2] Healey KD , Rifai AO, Maqueira AJ. et al. Hernia causing ureteral obstruction with hydronephrosis and subsequent urinary tract infection and sepsis. Cureus 2022;14:e29406. 36304363 10.7759/cureus.29406 PMC9586251

[3] Eilber KS , Freedland SJ, Rajfer J. Obstructive uropathy secondary to ureteroinguinal herniation. Rev Urol 2001;3:207–8. 16985719 PMC1476065

[4] Negmadjanov U , Daubert M, Rawlinson RD. et al. Laparoscopic repair of extraperitoneal ureteral inguinal hernia with mesh placement. Cureus 2020;12:e11067. 33224661 10.7759/cureus.11067 PMC7676819

[5] Feil N , Kwan D, Fateri C. et al. Case report of a pelvic kidney with ureteral obstruction from inguinal hernia entrapment and concurrent cryptorchid testis. J Educ Teach Emerg Med 2022;7:28–32. 10.21980/J8F345 PMC10332742 37465439

[6] Yahya Z , Al-Habbal Y, Hassen S. Ureteral inguinal hernia: an uncommon trap for general surgeons. BMJ Case Rep 2017;2017:bcr2017219288. 10.1136/bcr-2017-219288 PMC5353493 28275027

[7] Marcolin P , Poli M, de Figueiredo S. et al. Mesh repair versus non-mesh repair for incarcerated and strangulated groin hernia: an updated systematic review and meta-analysis. Hernia 2023. 10.1007/s10029-023-02874-0. 37679548

[8] Grice PT , Nkwam N. Inguinal hernia causing extrinsic compression of bilateral ureters leading to chronic obstructive uropathy. J Surg Case Rep 2018;2018:rjy062. 29644043 10.1093/jscr/rjy062 PMC5888468

[9] Ahmed S , Stanford R. Ureteric obstruction secondary to a paraperitoneal inguinal hernia. Ann R Coll Surg Engl 2016;98:e16–8. 26741680 10.1308/rcsann.2016.0024 PMC5210471

[10] Lebeau R , Traoré M, Anzoua KI. et al. Prognostic factors of postoperative morbidity and mortality of adult strangulated groin hernia. Indian J Surg 2016;78:192–6. 27358513 10.1007/s12262-015-1343-3 PMC4907908

